# Blood homocysteine concentration and mood disorders with mixed features among patients with alcohol use disorder

**DOI:** 10.1186/s12888-017-1342-y

**Published:** 2017-05-12

**Authors:** Francesco Oliva, Maurizio Coppola, Raffaella Mondola, Daniele Ascheri, Francesco Cuniberti, Gabriele Nibbio, Rocco Luigi Picci

**Affiliations:** 1 Department of Clinical and Biological Sciences, University of Turin, Regione Gonzole 10, 10043 Orbassano, TO Italy; 2 Addiction Department, Azienda Sanitaria Locale CN2, Alba, CN Italy; 3 Department of Mental Health, ASL CN1, 12037, Saluzzo, CN Italy; 4 Rita Levi Montalcini Department of Neuroscience, University of Turin, Turin, Italy

**Keywords:** Homocysteine, Hyperhomocysteinemia, Alcohol use disorder, Mood disorder, Mixed state

## Abstract

**Background:**

Blood homocysteine concentration (BHC) is higher in patients with alcohol use disorder (AUD). Previous studies have found a relationship between depressive symptoms severity and BHC in AUD patients and recently some authors have found high BHC among patients with bipolar disorder, both during manic and depressive episodes and in euthymic state. However, BHC in patients with mixed mood episode has not yet been investigated. The aim of this study was to evaluate the BHC of patients with AUD and mixed mood episode.

**Methods:**

A sample of AUD outpatients was assessed by Mini-International Neuropsychiatric Interview (MINI Plus): those with a DSM-IV-TR mood disorder with mixed features were included in the MIXED group (*n* = 45), whereas those without mood episode were gathered in the NO MOOD group (*n* = 23). Two subgroups, MIXMANIA and MIXDEPRESSION, were formed according to the prevalence of manic or depressive symptoms, assessed by Young Mania Rating Scale (YMRS), and Hamilton Rating Scale for Depression (HDRS). The Alcohol Use Disorder Identification Test (AUDIT) was used to appraise the AUD. BHC was determined by High-Performance Liquid Chromatography.

**Results:**

The MIXED group showed greater severity of both depressive (26.35 ± 9.96 vs. 4.77 ± 0.92; *p* < 0.001) and manic (22.35 ± 3.30 vs. 6.14 ± 1.12; *p* < 0.001) symptoms, and higher BHC (28.80 ± 11.47 vs. 10.83 ± 2.81; *p* < 0.001), than the NO MOOD group. BHC was strongly correlated to the HDRS, YMRS and AUDIT scores, just as HDRS was to YMRS, and AUDIT was to both HDRS and YMRS, in the MIXED group only (*p* < 0.001).

The MIXDEPRESSION subgroup showed higher BHC than the MIXMANIA subgroup (Mdn = 42.96, IQR = 10.44 vs. Mdn = 19.77, IQR = 5.93; *p* < 0.001).

A linear regression model conducted on the MIXED group found a significant predictive value for BHC of both HDRS (β = 0.560, *t* = 2.43, *p* = 0.026) and AUDIT (β = 0.348, *t* = 2.17, *p* = 0.044).

**Conclusions:**

Depressive symptoms seem to be mainly implicated in the BHC elevation among patients with both mixed features mood disorder and AUD.

## Background

Homocysteine (Hcy) is a sulphurated amino acid, derived from ingested methionine, that has received a great deal of attention for its role in many diseases, such as hypertension, cardiovascular disease and dementia [1, 2]. Hyperhomocysteinemia (HHcy) may be defined as a sustained elevation above normal concentrations of Hcy in plasma or serum, with the reference range being 7–15 μmol/l [3, 4]. Alcohol use disorder (AUD) is a problematic pattern of drinking, leading to clinically significant impairment or distress, characterized by behavioral and physical symptoms, such as withdrawal, tolerance, and craving [5].

Several authors since the early 1990s have studied the association between HHcy and AUD, reporting both that the increase of Hcy among AUD patients is proportional to AUD severity and that alcohol abstinence leads to a reduction of blood homocysteine concentration (BHC) [6–11]. Furthermore, some studies have reported a higher level of Hcy among those with AUD than among either controls or the general population, with measurements ranging from 13 to 120 μmol/l [6, 12–18].

At first, it was postulated that the HHcy found in patients suffering from AUD was due to a low intake of folate [15, 16], but succeeding studies discovered a complex interaction between alcohol, smoke, vitamin B6, vitamin B12, folate, betaine and Hcy metabolism that may involve poor absorption, hepatic uptake and retention of these nutrients, as well as both greater damage to the liver and an inhibition of enzyme activity [2, 19–25].

Although the correlation between folate deficiency and depression has been investigated since the 1960s [26, 27], studies that included measurements of BHC alongside vitamin status started in the 1990s [28, 29]. These studies not only found a significant association between HHcy and depression, regardless of vitamin status [29–33], but also suggested a poor response to antidepressants in patients with high Hcy levels [29]. According to the meta-analysis conducted by Almeida et al. [34], patients with HHcy are 70% more likely to have depression, while Refsum et al. [35] reported a doubling of the risk of having depression for Hcy levels >15 μmol/l. Although higher levels of Hcy are found even among patients with bipolar disorder [36, 37], no differences between euthymic and manic patients have been shown [38]. Conversely, the relationship between Hcy levels and mixed state has not yet been explored. Widely used definitions of mixed state classically refer to an affective condition in which depressive and manic features occur simultaneously [39–41]. Mixed state was studied in the past as a subtype of manic or depressive episode [42–45]; this concept was narrowly interpreted in DSM-IV, where the presence of both a full manic and a depressive syndrome for at least one week was required [46]. DSM-5 [5] has broadened the mixed state notion by introducing a mixed feature specifier that can be applied to both poles of bipolar disorder, as well as to major depressive episodes. Specifically, the specifier stresses the presence of at least three depressive symptoms in the manic pole and vice versa.

The link between Hcy and mixed features seems to have a plausible biological explanation: the Hcy functions as an excitatory amino acid and leads to an increase in glutammatergic neurotransmission, and thus to a calcium influx that has neurotoxic effects [47–51]; this could lead to instability in the affective symptomatology. It is noteworthy, albeit less well investigated, that increased serum Hcy levels have also been reported in patients with major depressive disorder characterized by an increase in anger and hostility, and even by psychotic symptoms [52–55].

Furthermore, major depressive disorder–AUD comorbidity is very frequent and seems to be more prevalent in mixed than in non-mixed episodes [46]. Similarly, only mixed episodes were found to be significantly associated with AUD symptoms in patients with a primary bipolar disorder [56].

Assuming the aforementioned relationship between mixed state, AUD and HHcy, we aimed to evaluate whether the BHC of AUD patients with mixed mood episode was higher than that of AUD patients without current episode of any mood disorder, and we also focused on the differences between mixed episodes with prevalent depressive symptoms and mixed episodes with prevalent manic symptoms.

## Methods

### Sample and procedures

All outpatients who accessed the Addiction Department of the *Azienda Sanitaria Locale CN2* (Alba, Cuneo, Italy) between January 2014 and November 2014 requesting treatment for AUD were invited to participate in this cross-sectional observational study. The eligibility criterion was a confirmed diagnosis of AUD under the DSM-IV definition. The exclusion criteria were (1) diagnosis of any current episode of mood disorder without mixed features (mania/hypomania or a major depressive episode); or (2) diagnosis of any other DSM-IV substance use disorder. Therefore, patients having a positive urine-screening test for substance abuse were also excluded.

An invitation was given at the end of the patient’s first visit and was accompanied by comprehensive information regarding the aims, methods, risks, and benefits of the study. If accepted, the agreement was formalized by the patient signing a written informed consent form. A unique identification code was assigned to each patient in order to maintain data anonymity and patient confidentiality. All patients agreeing to participate in the study were promptly assessed using the Mini-International Neuropsychiatric Interview (MINI Plus, Version 5.0.0), the Young Mania Rating Scale (YMRS), the Hamilton Rating Scale for Depression (HDRS), and the Alcohol Use Disorder Identification Test (AUDIT). At the first visit, all patients gave a blood sample for routine blood examination and a urine sample for urine toxicology screening. A measurement of total plasma Hcy concentration was included in the routine blood examination and was carried out through the improved High-Performance Liquid Chromatography (HPLC) method developed by Sawula et al. [57].

All patients with a DSM-IV mixed episode were then included in the MIXED group, and the others were allocated to the NO MOOD group. The MIXED group was subdivided into two subgroups (MIXMANIA and MIXDEPRESSION) according to the prevalence of either manic or depressive symptoms, as assessed by the Young Mania Rating Scale (YMRS) and the Hamilton Rating Scale for Depression (HDRS), respectively.

### Assessment tools

The MINI Plus Version 5.0.0 [58] was designed as a brief structured interview that could be used to diagnose DSM-IV Axis I psychiatric disorders. It has demonstrated its validity and reliability in comparison to other more complex and longer tools [59]. In the present study, the interview was administered to the enrolled patients in order: (1) to confirm the diagnosis of DSM-IV AUD; (2) to diagnose DSM-IV mixed episodes; (3) to exclude patients with any mood disorder without mixed features; and (4) to exclude patients with any other DSM-IV substance use disorder.

The AUDIT consists of a ten-item core questionnaire and an eight-item clinical procedure. The total score ranges from zero to 40, is based on the questionnaire alone, and gives a reliable estimate of the severity of alcohol use behavior [60]. The international and the Italian versions of this tool have both been verified for their good psychometric properties [61, 62]. In this study, the questionnaire was used as a measure of the severity of the AUD.

The HDRS [63] is a 17-item self-administered tool that is commonly used to evaluate the severity of depressive symptoms during a major depressive episode. The original five levels of severity have recently been reappraised among the depressed population by Zimmerman et al. [64], who compared the HDRS total score with the Clinical Global Impression severity index. The authors proposed the following four levels of severity: 0–7 for no depression, 8–16 for mild depression, 17–23 for moderate depression, and 24 or more for severe depression.

The YMRS [65] is an 11-item, clinician-administered tool that is commonly used to evaluate the severity of mania. Each item offers five levels of severity. The total score varies between zero and 60. This scale has shown good psychometric properties [65]. A score of 20 is usually used as a clinical threshold in randomized clinical trials evaluating the efficacy of antipsychotics on mania, although this has not yet been validated [66].

### Statistical analysis

The statistical analyses were performed using IBM SPSS Statistics for MACOS (Version 22.0. Armonk, NY: IBM Corporation).

Comparisons of the socio-demographic and clinical characteristics, both between the MIXED and the NO MOOD groups and between the MIXMANIA and MIXDEPRESSION groups, were carried out using Pearson’s χ2 test or Fisher’s exact test for the categorical variables, depending on the expected frequencies in each group. Comparisons of the continuous variables were performed using either Pearson’s independent-samples t-test or the Mann–Whitney U test, depending on whether the variables fell into normal or non-normal distributions, which was assessed using the Shapiro–Wilk test.

The association between the clinical scales (AUDIT, HDRS, YMRS) and the BHC was evaluated using Pearson’s r or Kendall’s Tau-b bivariate correlation coefficient, depending on the distribution of the variables. Finally, the clinical scales results correlated with BHC were evaluated as predictors for BHC among the MIXED group by a linear regression model using age, gender and the clinical scales as independent variables and the BHC as the dependent variable.

A *p*-value of 0.05 was used to designate statistical significance, but *p*-values resulting from multiple comparisons were adjusted using Bonferroni’s correction to control for the family-wise error rate.

## Results

Overall, 52 patients were asked to participate in the study. However, seven patients were excluded because they met at least one of the exclusion criteria (i.e., four patients had a major depressive episode without mixed features, one patient had an hypomanic episode without mixed features, and two patients had positive urine-screening test for cocaine). No patient refused to participate in the study.

As many as 23 out of the 45 enrolled patients (51.1%) had a DSM-IV mixed episode and were therefore included in the MIXED group, whereas 22 patients (48.9%) were not having any mood episode and so were included in the NO MOOD group.

**Fig. 1:**
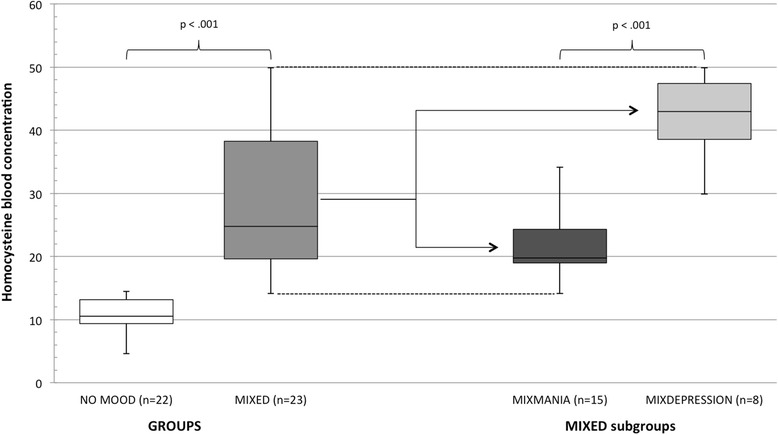
Box-plot of homocysteine blood level for the MIXDEPRESSION (*n* = 8) and MIXMANIA (*n* = 15) subgroups, the overall MIXED group (*n* = 23) and the NO MOOD group (*n* = 22)

The comparison between the MIXED and the NO MOOD groups did not show any statistically significant differences in socio-demographic characteristics or AUD severity, but it revealed a greater severity of both depressive and manic symptoms, and higher blood Hcy concentration, among the MIXED group (Table 1, Fig. 1). Moreover, in the NO MOOD group, the scores on both the mood scales were seen to be under the clinical threshold for severe disorder, i.e. < 20 for HMRS [66] and <24 for MDRS [64]; the BHC fell within the general population reference range, i.e. 7–15 μmol/l (Table 1 and Fig. 1).

**Table 1:** Socio-demographic and clinical characteristics of patients in the MIXED group (*n* = 23) vs. the NO MOOD group (*n* = 22)

	MIXED n (%)	NO MOOD n (%)	χ^2^ /F (df)	p
Sex^c^			.914 (1)	.339
male	16(69.6%)	18(81.8%)		
female	7(30.4%)	4(18.2%)		
Marital status^a^			1.08 (2)	.583
single	11(13%)	8(36.4%)		
married	9(47.8%)	12(54.5%)		
separated	3(39.2%)	2(9.1%)		
Education, years^b^			3.24 (3)	.369
1–5	1(4.3%)	2(9.31%)		
6–8	16(69.6%)	18(81.8%)		
9–13	5(21.7%)	1(4.5%)		
>13	1(4.3%)	1(4.5%)		
Employment^a^			.02 (1)	.894
unemployed	10(43.5%)	10(43.5%)		
employed	13(27.7%)	12(44.9%)		
	Mean(±SD)	Mean(±SD)	t/U (df)	p
Age^c^	45.9(±10.49)	45.8(±10.9)	.97(43)	.337
HDRS^d^	26.35(±9.96)	4.77(±0.92)	0(43)	< .001*
YMRS^d^	22.35(±3.30)	6.14(±1.12)	0(43)	< .001*
Homocysteine^d^	28.80(±11.47)	10.83(±2.81)	4(43)	< .001*
AUDIT^d^	24.57(±3.53)	22.18(±3.26)	191(43)	.036

^a^Pearson’s χ^2^ test

^b^Fisher’s exact test

^c^Student’s t-test

^d^Mann-Whitney U test

^*^Statistically significant, Bonferroni corrected *p* < 0.005

As regards the BHC for the two subgroups of the MIXED group, the MIXDEPRESSION subgroup had higher levels of Hcy than the MIXMANIA subgroup (Mdn = 42.96, IQR = 10.44 vs. Mdn = 19.77, IQR = 5.93; U = 1, *p* < .001; Fig. 1).

The bivariate correlation matrix built for each group showed that BHC was strongly correlated with the HDRS, YMRS and AUDIT scores in the MIXED group, just as HDRS was correlated with YMRS, and AUDIT was with both HDRS and YMRS (Table 2, *p* < .001). By contrast, no significant correlations were found in the NO MOOD group (Table 2).

**Table 2:** Bivariate correlation matrix for clinical variables in both the MIXED (*n* = 23) and the NO MOOD groups (*n* = 22)

MIXED^a^	Homocysteine	HDRS	YMRS	AUDIT
Homocysteine	1	.82*	.71*	.66*
HDRS		1	.78*	.59*
YMRS			1	.56*
AUDIT				1
NO MOOD^a^				
Homocysteine	1	−.26	.25	.03
HDRS		1	.22	.18
YMRS			1	−.14
AUDIT				1

^a^Kendall’s Tau-b correlation coefficient

*Statistically significant, Bonferroni corrected *p* < .017

All the clinical variables were strongly correlated in the MIXED group and thus all were included as predictors in the linear regression model. The model had a very high explanatory rating (adjusted R^2^ = 0.92, F = 54.3 [5, 17], *p* < .001), and it showed a significantly strong predictive value for BHC of both HDRS (β = 0.560, *t* = 2.43, *p* = .026) and AUDIT (β = 0.348, *t* = 2.17, *p* = .044).

## Discussion

The present study confirms that the BHC of patients with AUD and mixed episode is higher than that of patients with AUD who do not currently have mood episodes. The mean BHC among patients with AUD and mixed mood disorder was higher than that found in those suffering from euthymic bipolar disorder without AUD (28.8 ± 11.47 vs. 16.43 ± 10.4, [36]).

Moreover, the mean BHC in patients with AUD and no current mood episode was in line with that observed in the general population, and thus lower than that found in previous studies conducted among alcoholics, which made no distinction between those who had a mood disorder and those who did not [6, 12–18]. We might therefore suggest that HHcy is not in itself a condition related to AUD, but more exactly should be considered a common feature of patients with a co-occurrence of mood disorder and AUD.

As regards the relationship between clinical features and Hcy, the BHC was strongly correlated with the severity of depression, mania, and alcohol abuse only in patients with mixed features and, according to the findings of the linear regression, only the severity of depressive and AUD symptoms (and not that of manic symptoms) predicted blood Hcy concentration. Furthermore, as shown by Fig. 1, it seems that the two mixed state subgroups were well defined by blood Hcy levels: these were significantly higher among patients having prevalent depressive symptoms than in patients having prevalent manic symptoms.

Therefore, looking at the findings of the present study in the light of the results of previous studies, we might suggest that depressive symptoms seem to be mainly implicated in a raised BHC among patients with both a mixed features mood disorder and AUD. It might therefore be that this particular type of population could have a genetic vulnerability to suffer from both depression with mixed features and HHcy, and that this could be triggered by alcohol abuse severe enough to warrant a diagnosis of AUD. A similar correlation has been proposed by some studies concerning methylenetetrahydrofolate reductase (MTHFR) polymorphism among depressive and bipolar disorders [30, 67–69] .

The most important limitations of the present study were the sample size, which can provide enough power only for the primary aim (the comparison between the MIXED and the NO MOOD groups), and the absence of a group with major depressive disorder without mixed features (such a group would have allowed a more appropriate comparison with the previous literature about this type of population).

On the other hand, this is the first study evaluating BHC among AUD outpatients with a mood disorder with mixed features, and it suggests that further studies should be undertaken to investigate the possible predisposition of some AUD patients to suffer from mood disorders (especially those with mixed features) and HHcy, stressing also the possible role of the integration of nutrients (e.g. folate, vitamin B6, vitamin B12, betaine, etc.) in the treatment of mood disorders with AUD.

Future studies focusing on the assessment of the BHC of patients with bipolar disorder during the different stages of their illness are required, with close attention being given to whether or not AUD is present.

## Conclusions

To the best of our knowledge, this is the first study evaluating BHC among AUD outpatients having a mood disorder with mixed features and it suggests that HHcy is not in itself a condition related to AUD but that it should be considered a common feature of patients with a co-occurrence of mood disorder and AUD. Furthermore, depressive symptoms seem to be mainly implicated in the higher BHC among patients with both a mixed features mood disorder and AUD.

Further studies may be focused on the predisposition to develop HHcy in patients with comorbid mood and alcohol disorders.
